# Oncolytic virotherapy with human telomerase reverse transcriptase promoter regulation enhances cytotoxic effects against gastric cancer

**DOI:** 10.3892/ol.2021.13061

**Published:** 2021-09-22

**Authors:** Tomoya Kato, Mikihito Nakamori, Shuichi Matsumura, Masaki Nakamura, Toshiyasu Ojima, Hiroshi Fukuhara, Yasushi Ino, Tomoki Todo, Hiroki Yamaue

Oncol Lett 21: Article no. 490, 2021; DOI: 10.3892/ol.2021.12751

Following the publication of the above paper, the authors have requested that the article be retracted for the following reasons. First, a retraction was requested since a material transfer agreement (MTA) between the University of Tokyo and Wakayama Medical University had been violated upon submission of the article for publication. Secondly, the article had been submitted for publication without having received the final agreement of all the individual coauthors (in particular, the three coauthors HF, YI and TT) by prior written consent. Thirdly, the authors are uncertain that the results presented in Fig. 4B are fully reproducible, and therefore there is an element of uncertainty regarding the integrity of some of the data.

The Editor of *Oncology Letters* has agree to the authors’ request that this paper be retracted from the Journal. The Editor apologizes to the readership for any inconvenience caused.

